# Transmural migration of a gossypiboma: a rare cause of intestinal obstruction

**DOI:** 10.1590/0100-3984.2017.0201

**Published:** 2019

**Authors:** Isa Félix Adôrno, Rômulo Florêncio Tristão Santos, Andrea Cylene Tamura, Edson Marchiori, Thiago Franchi Nunes

**Affiliations:** 1 Universidade Federal de Mato Grosso do Sul (UFMS), Campo Grande, MS, Brazil.; 2 Universidade Federal do Rio de Janeiro (UFRJ), Rio de Janeiro, RJ, Brazil.

**Figure 1:**
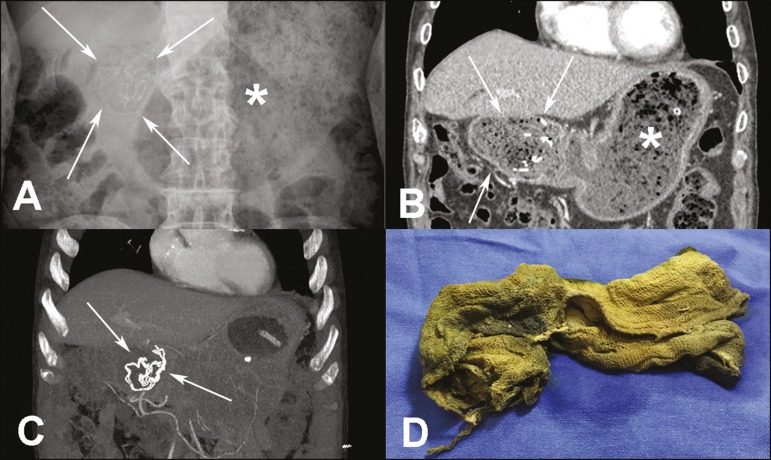
**A:** Conventional X-ray of the abdomen (with the patient in supine position) showing marked gastric distention (asterisk) and material containing hyperdense serpiginous lines in the region of the first portion of the duodenum (arrows). **B,C:** Contrast-enhanced coronal CT scan of the abdomen (**B**), with maximum intensity projection (**C**), confirming the X-ray findings and better characterizing the intraluminal mass in the duodenum (arrows) as heterogeneous, containing gas and dense linear structures, as well as showing thickening of the gastric wall and duodenal bulb. **D:** Surgical specimen showing a compress (gossypiboma) impregnated with biliary content (greenish-yellow coloring).

Dear Editor,

An 83-year-old man presented to the emergency department with an approximately one-month history of diffuse abdominal pain, nausea, bilious vomiting, and abdominal distension, the symptoms worsening in the last three days. He reported having lost 6 kg since the onset of symptoms. Six months prior, he had undergone cholecystectomy at another facility. On physical examination, the abdomen was slightly rounded, with increased bowel sounds, and was painful to superficial palpation in the mesogastrium. No organomegaly or palpable masses were observed. Upper gastrointestinal endoscopy showed gastric antral vascular ectasia, with a large amount of undigested food. The pylorus was lateralized, retracted, and stenotic, which precluded the passage of the endoscope into the duodenal bulb. An abdominal X-ray (Figure 1A) showed marked dilation of the gastric antrum, with an air-fluid level and serpiginous radiopaque areas in the duodenal region, characteristic of a foreign body (gossypiboma). An abdominal CT scan with intravenous contrast administration (Figures 1B and 1C) confirmed the X-ray findings and better characterized the intraluminal mass in the first portion of the duodenum, showing metallic wires within the mass and confirming upper gastrointestinal obstruction, as well as enhancement of the duodenal and gastric walls, probably due to an inflammatory reaction. There were no signs of pneumoperitoneum or cavitary fluid collections/abscesses. The patient underwent laparotomy, with laparoscopic suture closure of the duodenum and jejunostomy for feeding access. The presence of a foreign body (gossypiboma) was confirmed intraoperatively (Figure 1D). The gossypiboma, which was located in the first portion of the duodenum, resulted in gastric outlet obstruction and gastric dilatation.

Acute abdominal conditions have been the subject of various recent studies in the radiology literature of Brazil^(^^1^^-^^4^^)^. Gossypibomas have been identified in 0.02-0.1% of patients undergoing abdominal surgery^(^^5^^)^. Transmural migration of a gossypiboma is extremely rare. When it does occur, it is typically in the bowel, bladder, or chest. Spontaneous expulsion of a gossypiboma has been reported in only a few cases, the mean time from surgery to diagnosis being 2.2 years^(^^5^^,^^6^^)^.

Two types of reactions to foreign bodies have been described in the literature: fibroblastic and exudative. An aseptic fibrous response results in adhesion, encapsulation, and granuloma, usually remaining asymptomatic or causing chronic progressive symptoms over months to years. An exudative reaction causes the formation of a cyst or abscess that can establish fistulas to adjacent organs, the symptoms being more severe in such cases^(^^5^^,^^7^^)^. The increase in intra-abdominal pressure caused by a gossypiboma can result in partial or total necrosis of the intestinal wall^(^^6^^,^^7^^)^. The risk factors associated with the increase in the incidence of gossypiboma include emergency surgical procedures, prolonged surgical procedures, unplanned changes in the course of a procedure, the involvement of more than one surgical team, and a higher patient body mass index^(^^7^^)^.

The imaging findings preceding transmural migration of a gossypiboma are variable, depending on the nature of the sponge, its radiopaque marker, the length of time the foreign body has been present, and the type of reaction to it. A CT scan can reveal a poorly defined, heterogeneous mass, containing metallic wires and air, with a spongiform appearance. On contrast-enhanced CT scans, there can be edge enhancement, which is likely attributable to inflammation of the wall adjacent to the mass. A high-density capsule with a low density core is found in the majority of cases, making it difficult to distinguish between abscesses and hematomas. Calcification is a rare finding and is more common in long-standing cases^(^^5^^)^.
